# Characterization of the Biodistribution of a Silica Vesicle Nanovaccine Carrying a *Rhipicephalus (Boophilus) microplus* Protective Antigen With *in vivo* Live Animal Imaging

**DOI:** 10.3389/fbioe.2020.606652

**Published:** 2021-01-18

**Authors:** Karishma T. Mody, Bing Zhang, Xun Li, Nicholas L. Fletcher, Dewan T. Akhter, Sandy Jarrett, Jun Zhang, Chengzhong Yu, Kristofer J. Thurecht, Timothy J. Mahony, Neena Mitter

**Affiliations:** ^1^Queensland Alliance for Agriculture and Food Innovation, The University of Queensland, Brisbane, QLD, Australia; ^2^Animal Science, Queensland Department of Agriculture and Fisheries, St Lucia, QLD, Australia; ^3^Centre for Advanced Imaging (CAI) and Australian Institute for Bioengineering and Nanotechnology (AIBN), ARC Centre of Excellence in Convergent Bio-Nano Science and Technology and ARC Training Centre for Innovation in Biomedical Imaging Technology, The University of Queensland, St Lucia, QLD, Australia; ^4^Australian Institute for Bioengineering and Nanotechnology, The University of Queensland, Brisbane, QLD, Australia

**Keywords:** silica vesicles, adjuvants, Bm86 antigen, biodistribution, *in vivo* imaging, live animal imaging, immune response

## Abstract

Development of veterinary subunit vaccines comes with a spectrum of challenges, such as the choice of adjuvant, antigen delivery vehicle, and optimization of dosing strategy. Over the years, our laboratory has largely focused on investigating silica vesicles (SVs) for developing effective veterinary vaccines for multiple targets. *Rhipicephalus microplus* (cattle tick) are known to have a high impact on cattle health and the livestock industry in the tropical and subtropical regions. Development of vaccine using Bm86 antigen against *R. microplus* has emerged as an attractive alternative to control ticks. In this study, we have investigated the biodistribution of SV in a live animal model, as well as further explored the SV ability for vaccine development. Rhodamine-labeled SV-140-C_18_ (Rho-SV-140-C_18_) vesicles were used to adsorb the Cy5-labeled *R. microplus* Bm86 antigen (Cy5-Bm86) to enable detection and characterization of the biodistribution of SV as well as antigen *in vivo* in a small animal model for up to 28 days using optical fluorescence imaging. We tracked the *in vivo* biodistribution of SVs and Bm86 antigen at different timepoints (days 3, 8, 13, and 28) in BALB/c mice. The biodistribution analysis by live imaging as well as by measuring the fluorescent intensity of harvested organs over the duration of the experiment (28 days) showed greater accumulation of SVs at the site of injection. The Bm86 antigen biodistribution was traced in lymph nodes, kidney, and liver, contributing to our understanding how this delivery platform successfully elicits antibody responses in the groups administered antigen in combination with SV. Selected tissues (skin, lymph nodes, spleen, kidney, liver, and lungs) were examined for any cellular abnormalities by histological analysis. No adverse effect or any other abnormalities were observed in the tissues.

## Introduction

The beef cattle industry makes an important contribution to the global economy. In the recent years (2017–2018), it accounted for around 20% ($12.3 billion) of the total gross value of farm production. Ticks and tick-borne diseases have a serious impact on the global stock farming industry by negatively affecting the cattle health and production. Different approaches such as chemical acaricides and vaccines have been investigated to control cattle ticks. However, because of environmental and chemical-resistance issues associated with the use of acaricides, alternative solutions are required. One of the potential solutions is vaccination, which is increasingly preferred and is sought after to provide a long-term solution for more effective management of this pest.

The Bm86 antigen is a membrane-bound glycoprotein distributed mainly on the surface of the digestive tract of *Rhipicephalus* (*Boophilus) microplus* females and the principal component in cattle tick vaccine that can induce protective immune responses against this parasite. Compared with naturally induced immunity to *B. microplus*, the immune response elicited by Bm86 is regulated by host antibodies that interact with and damages (Willadsen and Bird, 1995). The recombinant Bm86-based tick vaccines have shown their efficacy for the control of cattle ticks, *B. microplus* and *Rhipicephalus annulatus* (Bastos et al., 2010; Sloat et al., 2010; Rodríguez-Valle et al., 2012). Therefore, it is crucial to develop more effective vaccine adjuvants/carrier that can elicit strong and sustained antibody responses against Bm86 antigen.

In the past decade, mesoporous silica nanoparticles (MSNs) have emerged as self-adjuvanting vaccine carriers with remarkable potential for the delivery of variety of antigens (Mody et al., 2013). MSNs are reported not only as biodegradable and biocompatible material but are also easy to modify and have low production costs. Our research over the last decade has focused on developing a platform technology using MSNs as antigen carriers and adjuvants for developing a nanovaccine with reduced number of injections, induction of long-term immune responses, and improved dose storage capacity. We have thoroughly investigated the potential of one class of MSNs, silica vesicles (SVs), as self-adjuvanting antigen carriers to develop successful vaccine delivery systems targeting bovine viral diarrhea virus-1 (mucosal disease) (Mahony et al., 2013, 2014; Mody et al., 2014a,b): *Anaplasma marginale* (anaplasmosis) (Zhang et al., 2016) and *Theileria parva* (East Coast Fever). In all of our studies, SVs successfully delivered the antigen of interest and initiated both humoral and cell-mediated immune responses. However, knowledge of the biodistribution and fate of this promising delivery technology remain unknown.

In this study, for the first time, we have investigated the biodistribution, fate, and immunogenicity of a single dose of SV adsorbed with Bm86 protein antigen. Using SV carriers and Bm86 antigen that were labeled with fluorescent makers and live animal (*in vivo*) and organ (*ex vivo*) imaging, we demonstrated the persistence of the carrier at the site of injection, whereas the antigen was continuously trafficked to the lymph nodes in the most immunologically responsive groups. This study provides important insights into the fate and safety of silica nanoparticles when used in vaccine applications. The study also suggests that these particles augment specific immune responses by acting as reservoir for continuous release of antigen.

## Materials and Methods

### Bm86 Production

A synthetic open reading frame for Bm86 antigen expressed in insect was assembled by GeneArt AG (Life Technologies) and cloned into pMK-RQ (kanR) using the cloning sites Sfil/Sfil. The purified plasmid DNA was transformed into *Escherichia coli* Top10 chemically competent cells (Invitrogen) to promote propagation and the transformed *E. coli* was cultivated overnight on LB agar plates containing 100 μg/mL ampicillin at 37°C. Single colonies conferring ampicillin resistance were selected and grown overnight in 10 mL of LB containing 100 μg/mL ampicillin with vigorous shaking at 37°C. Cells were harvested by centrifugation at 10,000 *g*, and pMK-RQBM86 vector was purified from the overnight culture using a Qiaprep spin miniprep kit (Qiagen). The 1890bp Bm86 fragment was released from the pMK-RQ vector by restriction endonuclease digestion, using *Bam*HI and *Hind*III restriction enzymes (NEB) following manufacturer's instructions. The Bm86 gene fragment was successfully ligated into the pFASTBAC1 expression vector and transformed into top 10 chemically competent cells (Invitrogen), followed by overnight cultivation at 37°C on LB plates containing 100 μg/mL ampicillin. The final plasmid construct was verified by DNA sequencing to confirm the correct insertion of the Bm86 open reading frame.

### Virus Seed Stock

Log-phase *Spodoptera frugiperda* S*f* 9 (ATCC® CRL-1711) cells were seeded at 1 × 10^6^ cells/well in Sf-900™ III SFM medium (Life Technologies, Scoresby, Victoria, Australia), in each well of a 6-well dish (Nunc, Life Technologies, Scoresby, Victoria, Australia), and allowed to adhere (1 h, 27°C). Bacmid DNA (1 μg) was added to prediluted ExpiFectamine™ Transfection Reagent (Life Technologies, Scoresby, Victoria, Australia) prepared according to manufacturer's instructions. Following incubation of the ExpiFectamine™/Bacmid DNA complex [room temperature (RT), 5 min], the DNA–lipid mixture was added dropwise onto the Sf9 cells and incubated for 72 h at 27°C. Once the cells appeared infected, the virus was harvested from the culture medium by centrifugation (500 g, 5 min). The cleared supernatant was transferred to a sterile tube and stored at 4°C, protected from light.

### Virus Amplification

Amplification of the Bm86 recombinant baculovirus was carried out using a shake flask format, with S*f* 9 cells seeded at 1 × 10^6^ cells/mL. An appropriate volume of the recombinant virus seed stock was used as inoculum. Both cell density and viability were monitored, and the culture supernatant was harvested when the cells were infected with the virus.

### Expression

The expression culture was set up using the following infection parameters. Briefly, 2.5 L of *Trichoplusia ni cells* High Five™ cells (Life Technologies, Scoresby, Victoria, Australia) was seeded at midlog phase in an Optimum Growth™ (Genesearch, Queensland, Australia) 5-L flask (135 rpm, 21°C) and infected with 1.5 × 10^6^ cells/mL of BM86 recombinant baculovirus. Cells were harvested at 120 h postinfection and separated from the supernatant by centrifugation at 9,000 g for 20 min.

### Purification

Supernatant from the expression culture was concentrated to 400 mL using a Hydrosart ultrafiltration cassette (Sartorius Australia Pty Ltd., Dandenong South, Victoria, Australia) 30-kDa nominal molecular weight cutoff. The concentrated supernatant was passed through a 0.45-μm filter (Merck Millipore, Bayswater, Victoria, Australia) and purified on an AKTA Pure FPLC System (GE Healthcare Life Sciences, USA) using immobilized affinity chromatography and purified under native conditions. Using a HiScale 26/20, 20 mL Ni Sepharose Excel (GE Healthcare Life Sciences, USA), the concentrated supernatant was loaded at a flow rate of 13 mL/min. To remove non–specifically bound proteins, the column was washed with 20 column volumes of wash buffer (20 mM sodium phosphate, 500 mM NaCl, 20 mM Imidazole, pH 8.0). The recombinant Bm86 protein was eluted in native elution buffer (five column volumes, 50% elution buffer, 20 mM sodium phosphate, 500 mM NaCl, 500 mM imidazole, pH 8.0, and five column volumes of 100% elution buffer). Fractions (2 mL) were collected during the elution steps and monitored by 10% sodium dodecyl sulfate–polyacrylamide gel electrophoresis (SDS–PAGE). Eluted fractions containing high-purity Bm86 were pooled and desalted into phosphate-buffered saline (PBS) using HiPrep 26/10 desalting columns (GE Healthcare Life Sciences, USA) following manufacturer's guidelines. The buffer exchanged samples were concentrated using an Amicon® Ultra-15 Centrifugal Filter with a 30-kDa molecular weight cutoff (Merck Millipore, Bayswater, Victoria, Australia). The sample was filter-sterilized with a 0.22-μm filter (Merck Millipore, Bayswater, Victoria, Australia) and stored at −80°C. An endotoxin test was carried out on the final sample using an Endosafe-PTS Limulus Amebocyte Lysate test kit (Charles River Laboratory Australia, Kilsyth, Victoria, Australia).

### Preparation of Rhodamine-Labeled SV-140-C_18_ and Cy5 Labeled Bm86 Antigen

The Rhodamine-labeled SV-140 (diameter 50 nm, wall thickness 6 nm, perforated by pores with an entrance size 16 nm and total pore volume of 0.934 cm^3^ g^−1^) were prepared and labeled as described in Mody et al. (2014a), Zhang et al. (2014), and Zhang et al. (2016). Cy5-labeled Bm86 (Cy5-Bm86) was prepared using the Abcam's manufacturer protocol (ab188288–Cy5 Fast Conjugation Kit).

### Adsorption of Cy5 Bm86 Antigen on Rho-SV-140-C_18_ and Western Hybridization

Adsorption reactions were set up as previously described (Mody et al., 2016), and the amount of unbound Bm86 protein was assessed by electrophoresis of the supernatants and the particles on SDS–PAGE gels (Supplementary Methods). Following SDS–PAGE electrophoresis, the Cy5-labeled Bm86 protein in the nanovaccine formulations was detected using Western blot hybridization as previously described (Zhang et al., 2016).

### Cellular Uptake of Cy5 Bm86 Antigen and Rho-SV-140-C_18_ Using Confocal Microscopy

Cy5-Bm86 was adsorbed on Rho-SV-140-C_18_ as described above. Murine macrophage-like RAW 264.7 cells (kindly donated by Dr. Barbara Rolfe from the Australian Institute for Bioengineering and Nanotechnology, The University of Queensland) were maintained in Dulbecco modified eagle medium supplemented with 10% fetal bovine serum at 37°C in a 5% CO_2_ incubator using standard cell culture procedures. For cellular uptake assays, 1 × 10^5^ RAW 264.7 cells were allowed to attach on sterile microscope coverslip chambers at 37°C for 24 h and then exposed to Cy5-Bm86 antigen adsorbed to Rho-SV-140-C_18_, Cy5-Bm86 antigen, Cy5 Bm86 antigen adsorbed to Rho-SV-140-C_18_ plus Montanide ISA 206 (Seppic), and Cy5-Bm86 antigen plus Montanide ISA 206, Rho-SV-140-C_18_. Montanide ISA 206 VG adjuvant was mixed with antigen following the manufacturer's instructions before exposing the mix to the cells. After 2-h incubation at 37°C and 5% CO_2_, the cells were washed three times with PBS pH 7.4. Cell membranes were stained bright green with wheat germ agglutinin, Alexa Fluor™ 488 Conjugate (WGA-488, Thermo Fisher Scientific), and nuclei were stained blue with Hoechst 33342 (Thermo Fischer Scientific), following the manufacturer's instructions. Cells were visualized using a 40× confocal laser-scanning microscope (LSM510METS, Zeiss). Microscopy was performed at the Australian Cancer Research Foundation (ACRF)–Institute for Molecular Bioscience Cancer Biology Imaging Facility, which was established with the support of the ACRF.

### Imaging and Immunization Studies Conducted in Mice

Adult BALB/c female mice were purchased from and housed in the Biological Resource Facility, The University of Queensland, Brisbane, Australia, under specific pathogen-free conditions. Eight-week-old female mice were housed in HEPA-filtered cages with 10 animals per group in an environmentally controlled area with a cycle of 12 h of light and 12 h of darkness. Food and water were given *ad libitum*.

The injectable doses were administered to investigate the induction of immune responses, and the treatment groups received injections as mentioned in Table 1. The mineral oil–based adjuvant Montanide-ISA206 was resuspended with the formulations of groups 3 and 5. One injection was administered at start of the experiment (day 0) to all the treatment groups except for the unimmunized group. Dose volumes of 100 μL (in 0.9% saline, Pfizer) were administered by subcutaneous injection at the tail base using a sterile 27-gauge needle (Terumo, Tokyo, Japan). Two animals were imaged and then sacrificed from each group on days 3, 8, and 13 after immunization. The remaining four animals were imaged and then sacrificed on day 28.

**Table 1:** Immunization groups used in the mice trial.

**Group**	**Number**	**Prototype vaccine/injection dose**
1	10	Rho-SV140-C_18_ (2,000 μg)
2	10	Cy5-Bm86 antigen (200 μg) adsorbed to Rho-SV-140-C_18_ (2,000 μg)
3	10	Cy5-Bm86 antigen (200 μg) adsorbed to Rho-SV-140-C_18_ (2,000 μg) + 50 μL Montanide ISA206
4	10	Unimmunized
5	10	Cy5-Bm86 antigen + 50 μL Montanide ISA206

*All doses (100 μL) were administered subcutaneously at the tail base*.

### *In vivo* and *ex vivo* Imaging of the Live Mice and Collected Organs

Images were acquired for ventral and dorsal views (supine and prone position) at each timepoint (3, 8, 13, and 28 days) for each group of mice. The same acquisition parameters for *ex vivo* imaging were used at for each timepoint. Fluorescent—560-, 580-, 600-, 620-, and 630-nm excitation, each 670 = nm emission, Auto exposure, 8 × 8 binning, f-stop 2, FOV 22.5 cm for Cy5, and for Rhodamine 520-, 540-, and 560-nm excitation, each 620-nm emission. Photography settings were as follows: 0.2-s exposure, no binning, f-stop 6, FOV 22.5 cm, X-ray—0.7-s exposure, f-stop 2, FOV 22.5 cm. Unmixing was used on all images, but made little difference in signal-to-noise ratio. This comparison is shown in the plot for the *ex vivo* data, as well as different image sets. Unmixing was done using the in-built function on Living Image (v4.7.2), and a model was created from an image of the samples and mouse before injection. Only background subtraction was applied to the *ex vivo* image sets, and no unmixing was applied to *ex vivo* image sets. Regions of interest were drawn around background zeroed organs, and the total radiant efficiency for each organ across a set of mice for each compound was normalized using the ratios of Cy5 intensity determined, and control organ was subtracted from the samples, averaged, and plotted with the standard deviation as the error.

Preimmunization blood samples were collected prior to the first injection and processed as previously described (Mody et al., 2014b). Blood samples postvaccination were collected via cardiac puncture at the respective timepoints. The animals were weighed and monitored for general health once a week. All the animals were assessed weekly, and they remained in good health throughout the duration of the study.

### Enzyme-Linked Immunosorbent Assay Protocol

The detection of total immunoglobulin G (IgG) Bm86-specific antibodies was performed by coating microtiter plates (96-well, Nunc, Maxisorb, Roskilde, Denmark) with Bm86 antigen solution (2 ng/μL, 50 μL per well) in PBS overnight at 4°C. The coating solution was removed, and the plates were washed once with PBS-T (PBS, 0.1% Tween-20, Sigma–Aldrich) and blocked with bovine serum albumin (5%, Sigma–Aldrich) and skim milk (5%, Fonterra, Auckland, New Zealand) in 200 μL PBS for 1 h with gentle shaking at RT. Plates were washed three times with PBS-T. Mouse sera samples were diluted from 1:100 to 1:6,400 in 50 μL PBS, and each dilution was added to the wells of the blocked plates followed by incubation for 2 h at RT. After washing, the mouse antibodies were detected using a horseradish peroxidase (HRP)–conjugated polyclonal sheep anti–mouse IgG antibodies (Chemicon Australia, Melbourne, VIC, Australia) diluted in PBS to 1:40,000, added to each well and incubated for 1 h at RT with gentle shaking. Plates were washed three times in PBS-T. TMB substrate (100 μL, Life Technologies) was added to each well and incubated for 15 min at RT; 100 μL of 1N HCl was added to the wells to stop the chromogenic reaction. The plates were read at 450 nm on the BioTek microplate reader (Winooski, US).

For the detection of IgG1 and IgG2a antibodies specific to Bm86 antigen, the following protocol was performed by coating microtiter plates (96-well, Nunc, Maxisorb, Roskilde, Denmark) with Bm86 antigen solution (10 ng/μL, 50 μL per well) in carbonate-bicarbonate buffer (0.1 M, pH 9.6) overnight at 4°C. The coating solution was removed, and the plates were washed once with PBS-T (PBS, 0.05% Tween-20, Sigma–Aldrich) and blocked with bovine serum albumin (2.5%, Sigma–Aldrich) in 200 μL PBS for 2 h with gentle shaking at RT. Plates were washed three times with PBS-T. Mouse sera samples were diluted from 1:3,200 to 1:6,400 in 50 μL PBS, and each dilution was added to the wells of the blocked plates followed by incubation for 1.5 h with gentle shaking at RT. Plates were again washed three times with PBS-T and the HRP-conjugated anti–mouse IgG1 and IgG2a antibodies (Chemicon Australia, Melbourne, VIC, Australia) diluted in PBS to 1:5,000, added to each well, and incubated for 1 h at RT with gentle shaking. Plates were washed three times in PBS-T. TMB substrate (100 μL, Life Technologies) was added to each well and incubated for 15 min at RT; 100 μL of 1 N HCl was added to the wells to stop the chromogenic reaction. The plates were read at 450 nm on the BioTek microplate reader (Winooski, US).

*T* test (Giuliano et al., 1999) was used to calculate the mean values of total IgG, and the graph was plotted at two dilutions 3,200 and 6,400, similarly for IgG1 and IngG2, a mean value was plotted for 6,400 dilution.

### Histopathology

Heart, kidney, lymph node, lungs, liver, and injection sites from the sacrificed mice were collected and fixed in 10% formalin for 48 h. The organs were further processed and embedded in paraffin, and 8-μm sections were cut using the Leica RM 2245 Rotary Microtome. The sections were then stained using the following hematoxylin–eosin staining procedure. Sections were first dewaxed in xylene (three changes of 2 min each) and then rehydrated in absolute alcohol (two changes of 2 min each), in 90% for 2 min, in 70% for 2 min, then washed in running tap water for 2 min and stained in hematoxylin for 3 min, and again washed in running tap water for 2 min. Sections were then washed in 70% alcohol for 2 min and stained in eosin for 3 min. Sections were then washed in 95% alcohol for 2 min and then in absolute alcohol (three changes of 2 min each). Finally, the sections were rapidly dehydrated and fixed in xylene (three changes of 2 min each) and mounted in DePeX. The sections were then observed under the Zeiss LSM 510 META confocal microscope at 20× magnification.

## Results

**Figure 1:**
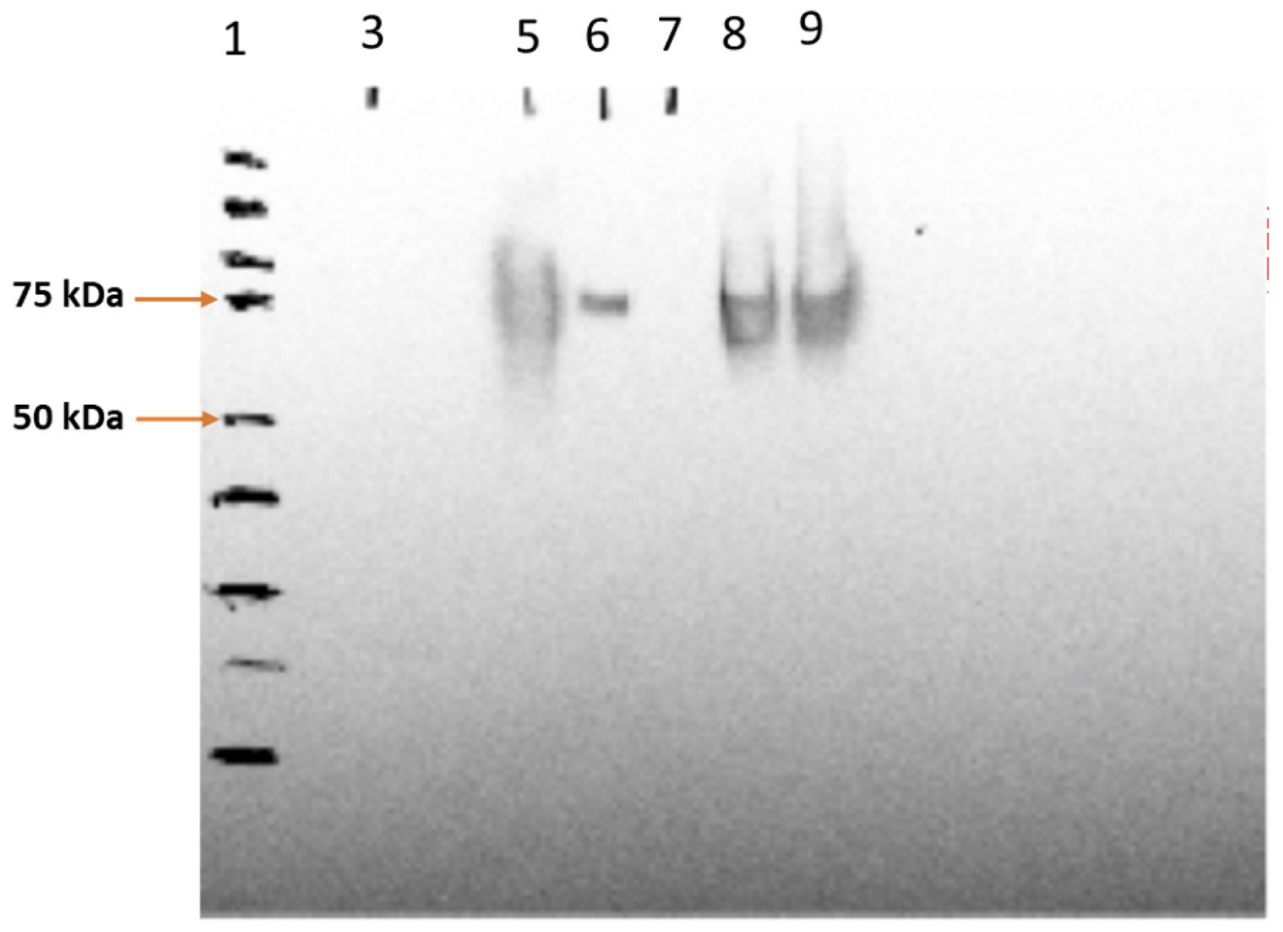
Western blotting analysis of Cy5-Bm86 in the vaccine formulations, lane 1—protein ladder, lane 5—Cy5-Bm86 protein, lane 7—Cy5-Bm86/ Rho-SV-140-C_18_ Supernatant, lane 8—Cy5-Bm86/Rho-SV-140-C_18_ pellet (first antibody 869 polyclonal sheep 1:5,000; second antibody monoclonal anti-goat/sheep 1:10,000).

The SV-140-C_18_ particles investigated in this study have been characterized previously (Mody et al., 2016). In this study, the 100 μg of Cy5-Bm86 protein was adsorbed to 1 g of the Rho-SV-140-C_18_ vesicles. The SDS–PAGE analyses on the adsorption samples demonstrated that the integrity of Bm86 protein was preserved postlabeling (Supplemental Figure 1). Furthermore, Western blot analysis of the fluorescently labeled nanovaccine formulation demonstrated the Cy5-labeled Bm86 retained its native antigenicity as it was recognized by the antibodies in serum from a sheep immunized with the unlabeled antigen from a previous study (Figure 1).

### Cellular Uptake

**Figure 2:**
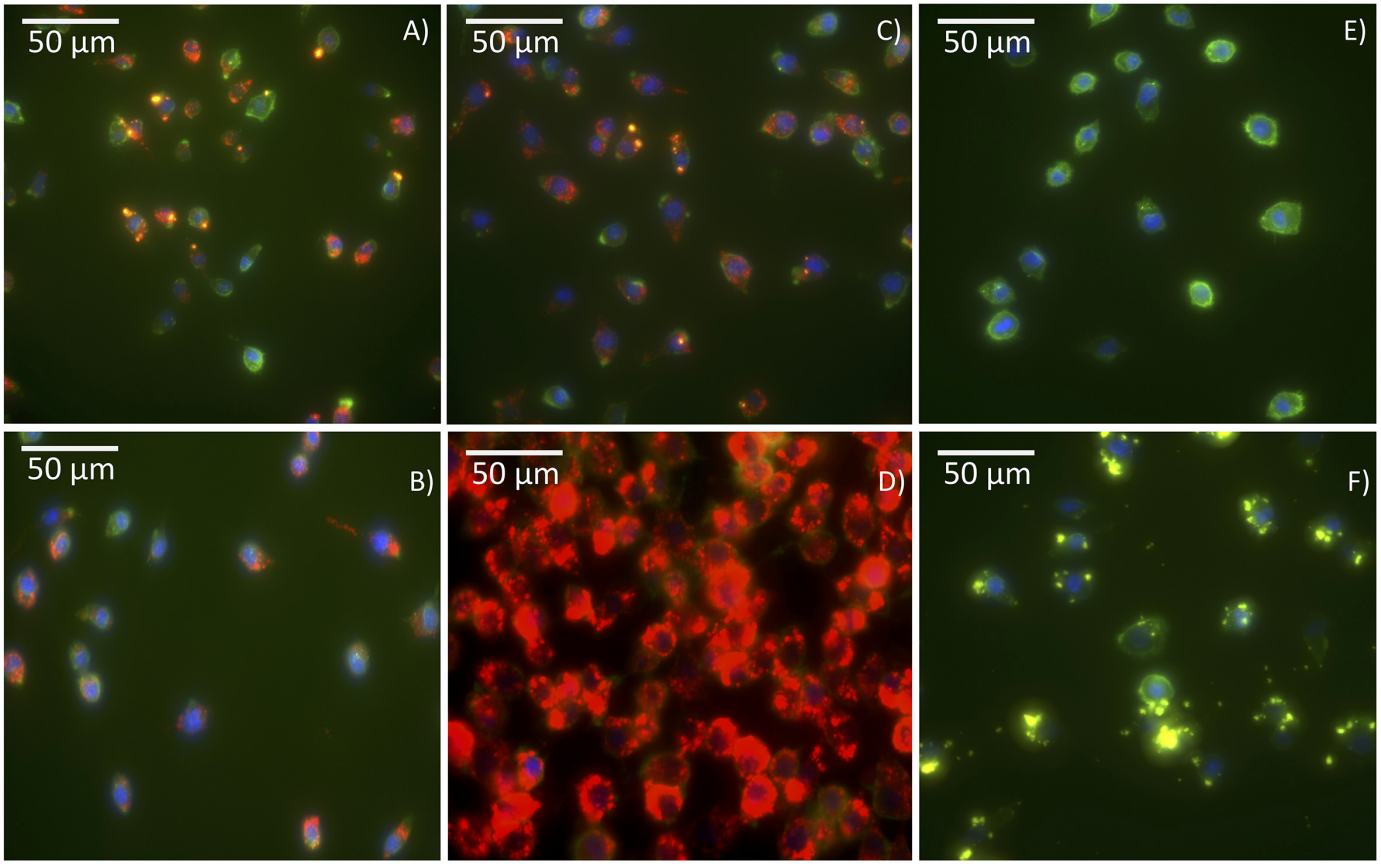
Cellular uptake Confocal fluorescence microscopy of RAW 264.7 cells following incubation with different treatments. The images are of **(A)** Cy5-Bm86 /Rho-SV-140-C_18_, **(B)** Cy5-Bm86 antigen, **(C)** Cy5-Bm86 antigen/Rho-SV-140-C_18_ + Montanide ISA206, **(D)** Cy5-Bm86 antigen + Montanide ISA206, **(E)** cells alone and **(F)** Rho-SV-140-C_18_. Cell membranes were stained bright green with wheat germ agglutinin, Alexa Fluor™ 488 Conjugate (WGA-488) and nuclei were stained blue with Hoechst 33342. Cy5 is represented in red and Rhodamine is represented in yellow and the overlap of Rhodamine and Cy5 appears orange due to an overlap of red and green.

The cellular uptake of the antigen Cy5-Bm86 adsorbed to Rho-SV-140-C_18_ vesicles was investigated using the murine macrophage cell line RAW 264.7 cells and confocal microscopy. In order to study the uptake and intracellular fate of the labeled antigen and SVs, cell membranes were stained bright green with wheat germ agglutinin, and nuclei were stained blue with Hoechst. Cy5 is represented in red, and Rhodamine is represented in yellow; the colocalization of antigen with SVs appears orange because of the overlap of red and yellow. As illustrated in the confocal images (Figure 2), labeled antigen and SVs were effectively taken up by the cells as shown by the presence of fluorescence inside the cells compared to the control group (Figure 2F). Cy5-Bm86 plus Montanide ISA 206 adjuvant and Cy5-Bm86–loaded SV-140-C_18_ plus Montanide ISA 206 showed considerably higher fluorescence intensity in the cells compared to other groups [control (F), Cy5-Bm86 (B), and Cy5-Bm86 loaded SV-140-C_18_ (C)]. Moreover, both the red and yellow fluorescence overlay to give an orange color in the merged images for Cy5-Bm86–loaded SV-140-C_18_, as well as Cy5-Bm86–loaded SV-140-C_18_ plus Montanide ISA 206, indicated that both Cy5-labeled antigen and Rho-labeled SV were taken up by the RAW 264.7 cells.

### *In vivo* Biodistribution Studies in Live Mice

**Figure 3:**
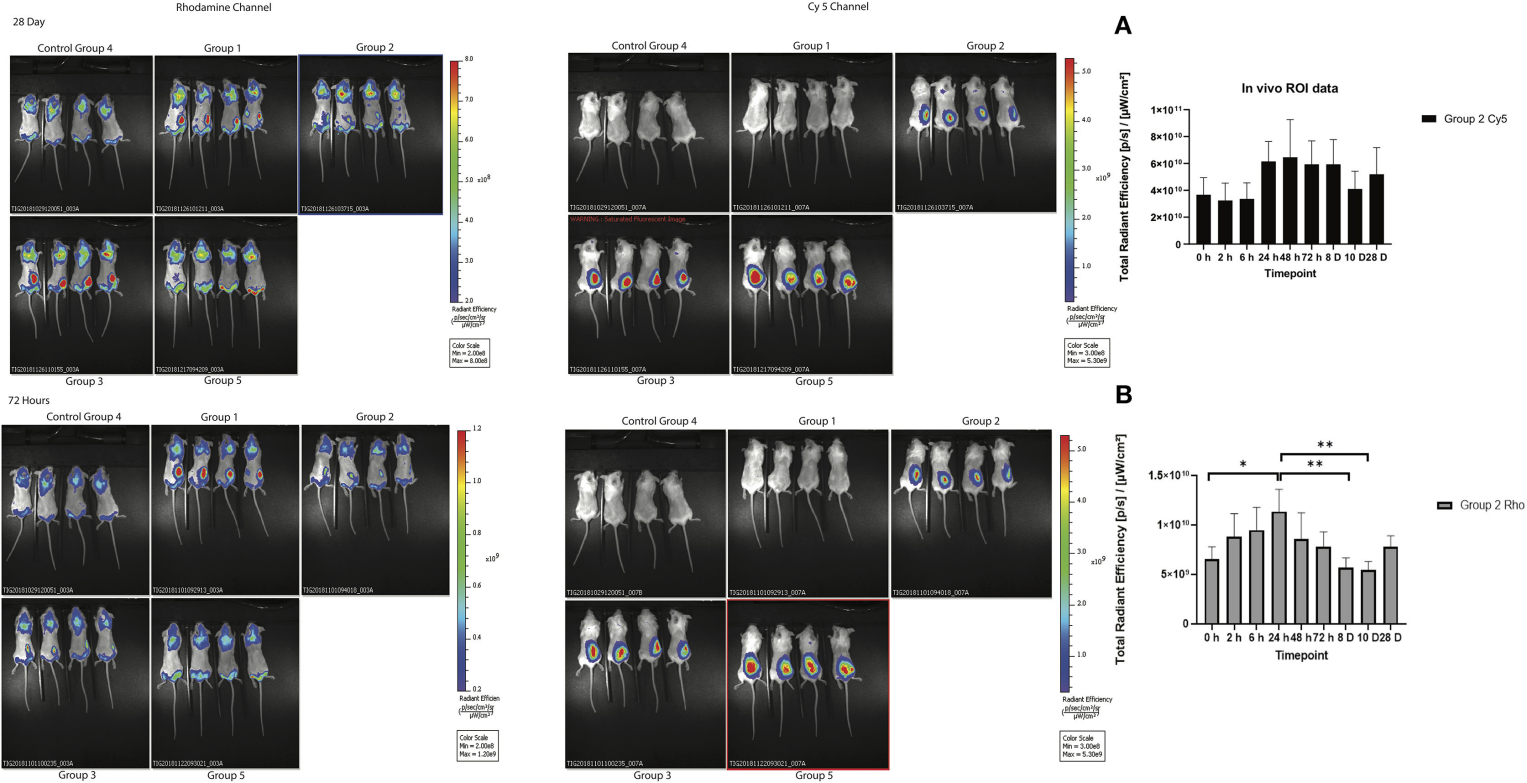
*In vivo* biodistribution of the fluorescently immunogens from at 72 h and 28 days of BALB/c mice injected with group 1: Rho-SV140-C_18_ (2,000 μg); group 2: Cy5-Bm86 antigen (200 μg)/Rho-SV140-C_18_ (2,000 μg); group 3: Cy5-Bm86 antigen (200 μg) loaded on Rho-SV140-C_18_ (2,000 μg) + 50 μL Montanide ISA206; group 4: unimmunized; and group 5: Cy5-Bm86 antigen + 50 μL ISA206. **(A)** *In vivo* total radiant efficiency of group 2 animals in Cy5 channel (no significant difference observed between the different timepoints when assessed by Tukey multiple comparison test. **(B)** *In vivo* total radiant efficiency of group 2 animals in Rhodamine (Rho) channel * <0.05, ** <0.01 as assessed by Tukey multiple-comparisons test.

To track the nanoparticles and antigen *in vivo*, whole-animal fluorescence imaging was undertaken. To assess the potential efficacy of antigen as well as SV as adjuvants, the biodistribution of both nanoparticles and antigen was evaluated at several timepoints after one subcutaneous immunization. Whole-animal fluorescence imaging (Figure 3) clearly demonstrated that most of the Rho-SV-140-C_18_ and antigen remained at the site of injection, with no obvious decrease in fluorescence intensity, for the duration of the experiment to day 28.

### Generation of Total IgG, IgG1, and IgG2a Antibodies in Response to Bm86 Antigen

**Figure 4:**
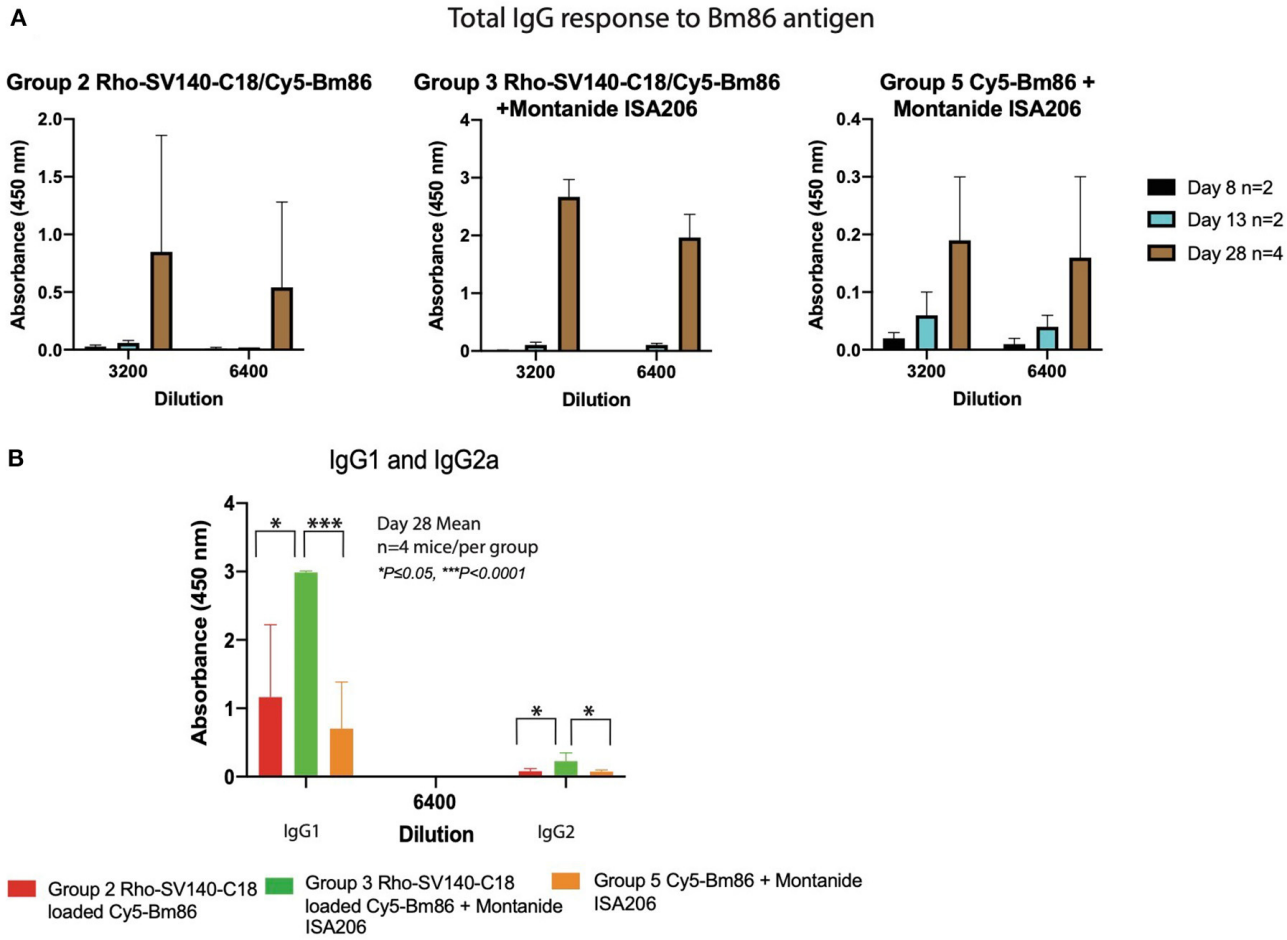
Bm86-specific antibody responses in mice at different timepoints. **(A)** Mean values of total IgG responses of groups 2, 3, and 5 [day 8 (*n* = 2), 13 (*n* = 2), and 28 (*n* = 4)] animals and **(B)** mean value of IgG1 and IgG2a response at 6,400 dilution day 28 (*n* = 4) after one subcutaneous immunization. Error bars represent one standard deviation. ^*^P < 0.05, ^***^P < 0.001.

To evaluate the capacity of the Cy5-Bm86/Rho-SV-140-C_18_ and Cy5-Bm86/Rho-SV-140-C_18_ plus Montanide ISA 206 nanovaccine formulations to elicit immune responses, animals were immunized with single subcutaneous injections. The total IgG responses of the immunized mice were analyzed by anti-Bm86–specific enzyme-linked immunosorbent assays with sera harvested at days 3 (*n* = 10), 8 (*n* = 8), 13 (*n* = 6), and 28 (*n* = 4). The animals in all the treatment groups remained healthy and in the normal-weight range throughout the trial period (data not shown). Four showed similar responses on day 13. However, group 3 induced strong antibody responses at day 28 timepoint, and less mouse-to-mouse variation was observed with the animals in this group (average OD value of 1.97 ± 0.37 at 6,400 dilution) (Figure 4). The average OD values for groups 2 and 5 were 0.54 ± 0.74 and 0.17 ± 0.16, respectively, at the 6,400 dilution. The mice receiving the Rho-SV-140-C_18_ only (group 1) and the unimmunized group (group 4) showed no detectable Bm86-specific antibody responses. A stronger IgG1 response was detected in the mice (*n* = 4) of group 3 on day 28 compared with the animals in groups 2 and 5. Low levels of IgG2a-specific response to Bm86 antigen was also observed in group 3 animals (Figure 4).

### *Ex vivo* Imaging of the Collected Mice Organs

**Figure 5:**
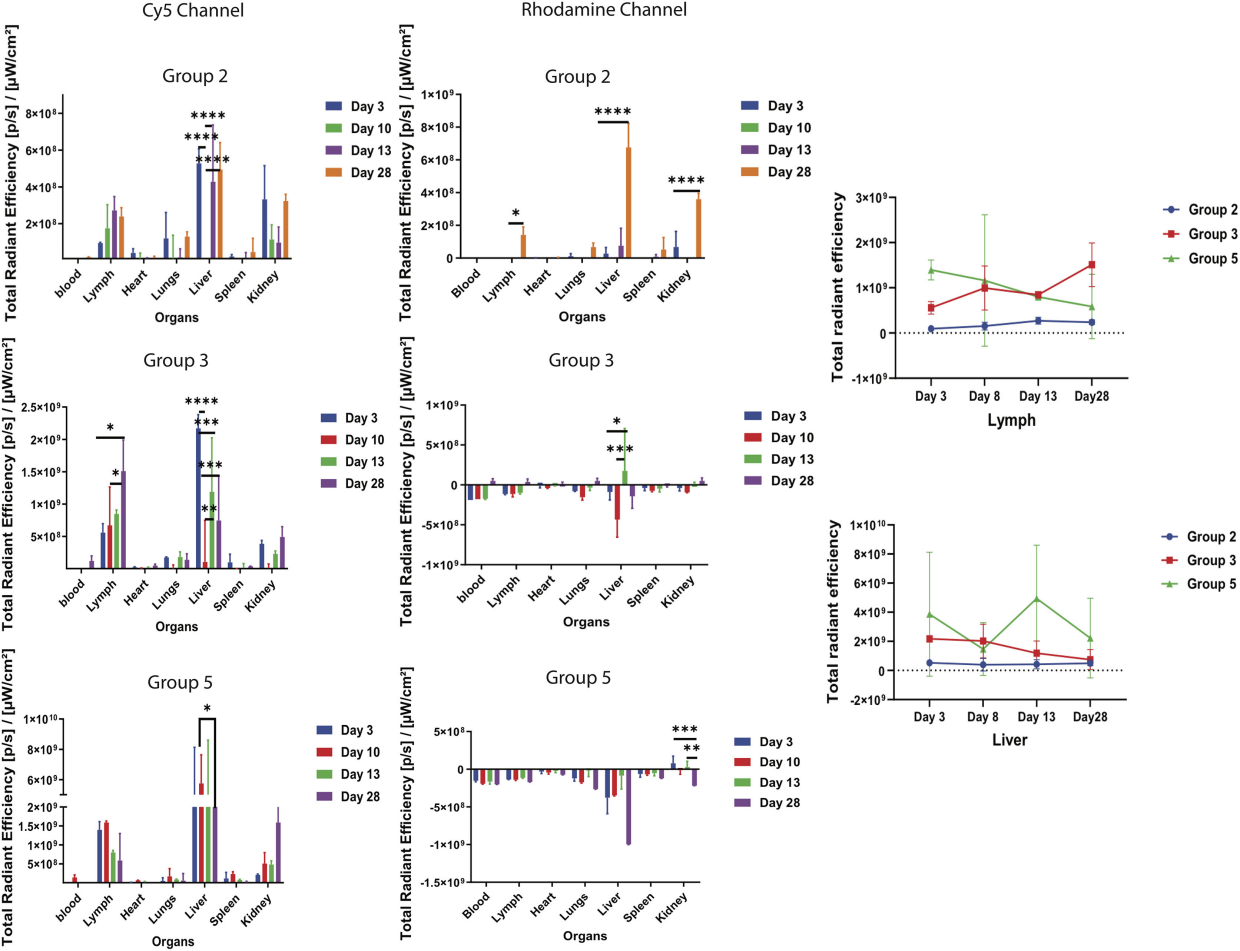
*Ex vivo* fluorescent imaging of mouse organs. The graph represents normalized quantitative Cy5 fluorescence and Rhodamine intensity in each organ at different timepoints. *P < 0.5, **P < 0.01, ***P < 0.001, ****P < 0.0001. Lymph and liver samples were collected at days 3, 8, 13, and 28 postvaccination.

To track the movement of the Cy5-Bm86 and Rho-SV-140-C_18_ blood, lymph, heart, lungs, liver, spleen, and kidneys were collected from the sacrificed animals at days 3, 8, 13, and 28 postvaccination. Weak Rhodamine intensities were too low to accurately determine away from the injection site of the *ex vivo* images. Cy5 fluorescent intensity as observed in the Figure 5 was the highest recorded in the lymph nodes on day 28 of group 3 animals, followed by the groups 5 and 2 animals. Trafficking of the antigen to the lymph nodes were observed in groups 3, 2, and 5 mice. No significant difference was observed in the blood, heart, lungs, liver, spleen, and kidney samples in all the treatment groups compared to the control group. *Ex vivo* fluorescence imaging (Supplementary Figure 5) on the excised organs of group 3 mice (*n* = 2, at each timepoint) on days 3, 8, and 28 showed a large amount of Cy5 signal at the site of injection and lymph node compared to the mice (*n* = 2) in the control group.

### Histopathology of Tissue Organs From Immunized Mouse

**Figure 6:**
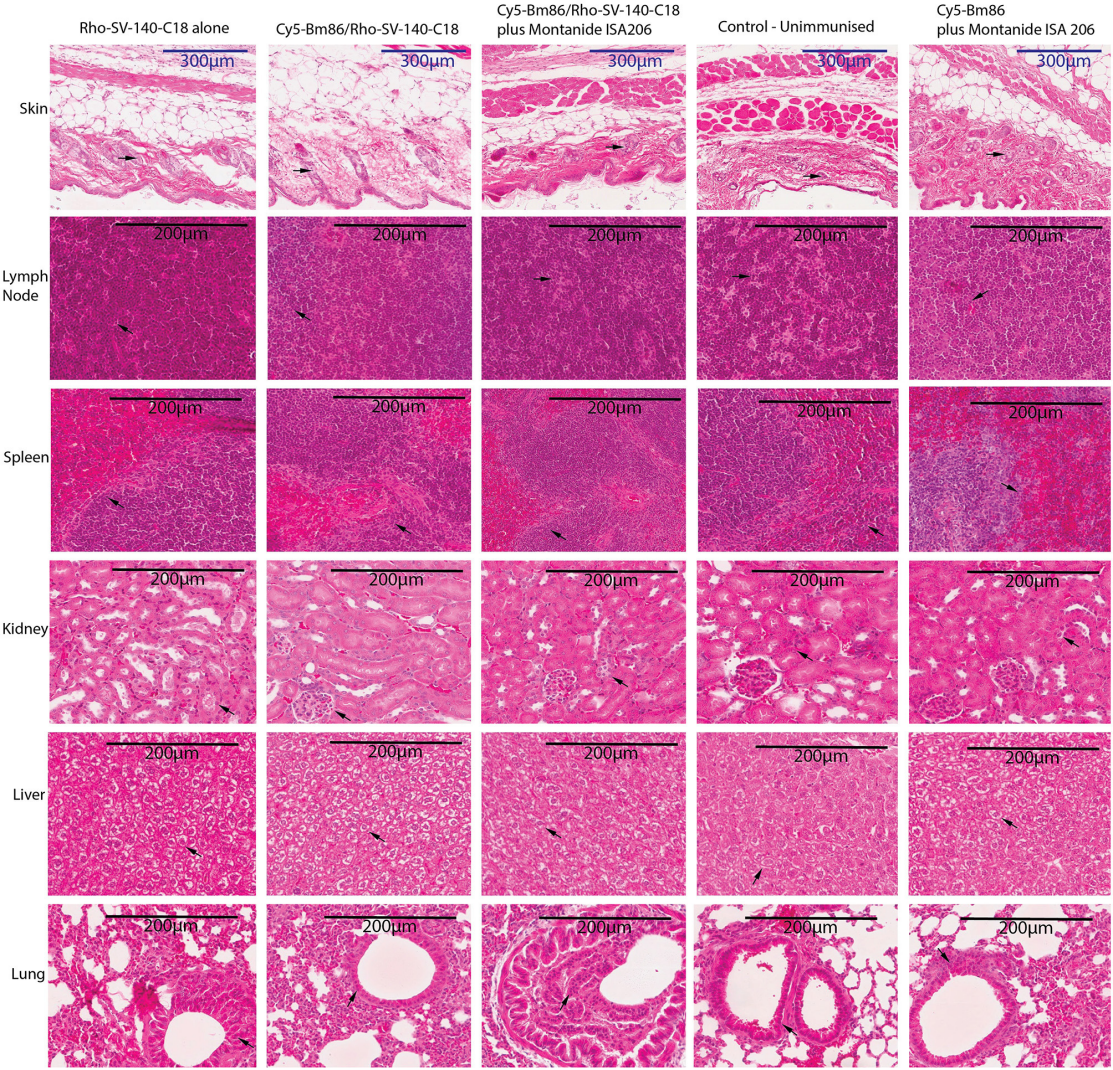
Histopathology studies of tissue organs from a mouse injected with nanovaccine immunizations. At the end of the experiment, organs fixed in formalin were harvested from two mice for each treatment group and embedded in paraffin; sections were stained with hematoxylin–eosin stain and imaged at 20× magnification; skin-injection site, lymph node, spleen, kidney, liver, lung. The arrows on the figure suggest that no obvious differences were observed at cellular levels in the organs of the treatment groups vs. the control unimmunized group.

Mouse organ and tissue samples were collected from all the treatment groups at day 28 postvaccination. Histopathology assessments of sections from skin-injection site, lymph node, spleen, kidney, liver, and lung demonstrated that administration of Cy5-Bm86/Rho- SV-140-C_18_ (group 2), Cy5-Bm86/Rho- SV-140-C_18_ plus Montanide ISA 206 (group 3), Cy5-Bm86 plus Montanide ISA 206 (group 5), and SV-140-C_18_ alone (group 1) did not have any observable detrimental effects when compared to similar tissues from unimmunized control mice (group 4). Briefly, in all of the organ sections, the cells of the treatment group looked similar to the unimmunized control group. No granulomas were formed at the site of injection when injection site samples were collected, and there is no evidence of inflammation at the injection site of treatment groups as confirmed by histopathology. There was no evident change in the thickness of the skin layer relative to the control group. In the spleen samples, there were no overt changes in the red-to-white pulp ratio compared to the control group. Similarly, the kidney and liver samples collected from the animals given the treatment did not show changes in the blood vessels and shrinkage or collapse of the blood vessels. Lungs have a variable morphology, and no signs of cell death, inflammation, or major difference was observed in the treatment groups compared to the unimmunized control. In the lymph nodes, no evident signs of infiltration or cell death was observed between the treatment groups and the control group (Figure 6).

## Discussion

For subunit vaccines to be successful commercially, it is important that they elicit strong and specific immune responses and that any adjuvant used are safe (Jin et al., 2019). In our recent nanoparticle-based vaccine development studies, we have reported the capacity of SVs as efficient nanocarriers for adsorption of different antigens targeting varied animal diseases (Mody et al., 2014b, 2015; Zhang et al., 2016; Zhao et al., 2016). In these studies, the SV-140 materials displayed an excellent capacity to act as self-adjuvanting delivery system. The promising results from SV-140 materials encouraged us to track their biodistribution to ensure that they are not getting accumulated inside the mice organs, a requisite for the development of a successful subunit vaccine.

The cellular uptake of Bm86 antigen conjugated with Cy5 (Cy5-Bm86) and Rho-SV-140-C_18_ vesicles was first investigated using confocal microscopy in RAW 264.7 cells. Cy5-Bm86 plus Montanide ISA 206 adjuvant and Cy5-Bm86 adsorbed to SV-140-C_18_ plus Montanide ISA 206 showed considerably higher fluorescence intensity in the cells compared to other groups. MSN surface chemistry plays a critical role in influencing the cellular uptake characteristics. Nanoparticle size affects the *in vivo* pharmacokinetics and its cellular interaction (Hoshyar et al., 2016) Our cellular uptake assay demonstrated that the fluorescently labeled antigen and the SVs were evenly taken up by live cells. Therefore, these formulations were further investigated in an animal trial. This research work for the first time highlights and tracks the biodistribution characteristics of Rho-SV-140-C_18_ and of Cy5-Bm86 antigen using live and *ex vivo* imaging of BALB/c mice that were administered one dose of vaccination for up to 28 days.

As highlighted by Li et al. (2010), biodistribution changes are dependent on nanoparticle properties and interactions with the living system (Li et al., 2010). In particular, nanoparticle size affects the biodistribution of nanoparticles throughout the body (Sonavane et al., 2008; Choi et al., 2011; Dreaden et al., 2012). Most of the studies in the literature have shown that size of the particles has an impact on their biodistribution characteristics; the microparticles remain in the body much longer than nanoparticles (Faraji and Wipf, 2009). In this study, most of the SVs are deposited at the site of the subcutaneous injection and did not get concentrated in different mouse organs. Faraji and Wipf (2009) also reviewed that microparticles remained in the mouse spleen and the site of injection for at least 2 weeks, whereas nanoparticles of the same material were almost completely cleared in the same time frame (Faraji and Wipf, 2009). The largest accumulations of nanoparticles typically occur in the blood, liver, and spleen (Dreaden et al., 2012). Our results have shown the 50 nm Rho-SV-140-C_18_ was mostly retained at the site of injection, and minor signal was observed in the liver, spleen, and kidneys when the nanoparticles were loaded with antigen. As discussed by Yu et al. (2012), the silica nanoparticles were likely internalized by the reticuloendothelial system following physical sequestration in the liver and spleen (Yu et al., 2012). In the control group injected with only Rho-SV-140-C_18_, there was no accumulation of the nanoparticles in liver, spleen and kidneys.

Literature suggests that silica nanoparticles when administered via intravenous route were actively excreted by the mice within a few days, mainly by the urinary tract but also within feces, due to particle excretion through the biliary route (Malfatti et al., 2012). However, additional studies have shown that the silica nanoparticles can be retained in the body for longer periods of time and can be detected in liver and spleen up to 8 weeks postinjection (Cho et al., 2009; Huang et al., 2011; Malfatti et al., 2012). The biodistribution and the excretion rate of silica nanoparticles are greatly dependent on particle size (Cho et al., 2009; He et al., 2011; Yu et al., 2012; Fu et al., 2013), the particle shape (Decuzzi et al., 2010; Huang et al., 2011; Yu et al., 2012), and perhaps the gender of the animal (De Jong et al., 2013). To our knowledge, there no studies that have investigated the biodistribution properties of SVs when administered subcutaneously; our study highlights that SVs were retained at the site of injection and did not get concentrated to major organs of mice. Further studies are necessary to evaluate the biodistribution of the SVs for longer duration in order to determine if their long-term fate differs from the current study.

The presence of Cy5 signal associated with the Bm86 antigen in the lymph nodes and spleen over multiple weeks is a strong indicator of the capacity of SVs to enable slow release of the antigen and to successfully deliver the antigen to elicit immune responses. The Cy5-Bm86/Rho-SV-140-C_18_ in combination with Montanide adjuvant ISA206 induced highest antibody responses, followed by the group injected with Cy5-Bm86/Rho-SV-140-C_18_ after a single subcutaneous immunization. It is quite evident that stronger IgG1 response was elicited by the group 3 animals at the day 28 timepoint compared to the groups 2 and 5 animals, similarly some IgG2a response was noticed in the group 3 animals compared to group 2 and 5. This is a strong indicator that a single dose of Cy5-Bm86/Rho-SV-140-C_18_ in combination with Montanide adjuvant ISA206 generated a stronger (Th2) antibody response against Bm86 antigen. These results demonstrate the potential of SV-140 as both efficient vaccine delivery vehicles and potent adjuvants, in agreement with a previous study (Mody et al., 2014b).

In summary, we have demonstrated that Cy5-Bm86/Rho-SV-140-C_18_ plus adjuvant ISA206 were efficiently taken up by murine macrophages. In addition, this formulation elicited strong antibody responses after a single subcutaneous immunization, and most of the Rho-SV-140-C_18_ remained at the site of injection and slighted traced in the liver and spleen of groups 2 and 3, but mostly the SVs were not trafficked to other major organs of the mice by day 28. These findings provide strong evidence that SV-140 nanomaterials were efficiently taken up by the cells, but also actively act as carriers and adjuvants by engaging and mediating in the immunology pathways to induce essential immune responses. Importantly, this study suggests the SV-140 nanomaterials are a safe vaccine delivery platform. Furthermore, strong immunological responses were detected, and no pathological changes were observed at the injection site or the major organs, thus further proving the potential of SVs as a promising new generation vaccine adjuvant and delivery vehicle for the development of safe and effective veterinary subunit vaccines.

## Data Availability Statement

The original contributions presented in the study are included in the article/supplementary materials, further inquiries can be directed to the corresponding author/s.

## Ethics Statement

All procedures were approved by The University of Queensland, Ethics Committee (approval no. QAAFI/CAI/089/18), as required by the Animal Care and Protection Act (2001) and The Australian Code for the Care and Use of Animals for Scientific Purposes (eighth edition).

## Author Contributions

KM, BZ, XL, NF, DA, CY, KT, TM, and NM designed research. KM, BZ, XL, NF, DA, SJ, and JZ performed research. KM, BZ, XL, NF, and DA analyzed data. KM, BZ, XL, NF, DA, SJ, JZ, TM, and NM wrote the paper. All authors contributed to the article and approved the submitted version.

## Conflict of Interest

The authors declare that the research was conducted in the absence of any commercial or financial relationships that could be construed as a potential conflict of interest.
