# Supplementary material for: Characterization of the Biodistribution of a Silica Vesicle Nanovaccine Carrying a Rhipicephalus (Boophilus) microplus Protective Antigen With in vivo Live Animal Imaging
Source: Front Bioeng Biotechnol. 2021 Jan 18;8:606652. doi: 10.3389/fbioe.2020.606652 (PMC7848120; doi:10.3389/fbioe.2020.606652)
Supplement: Supplementary file 1 [file Data_Sheet_1.docx]

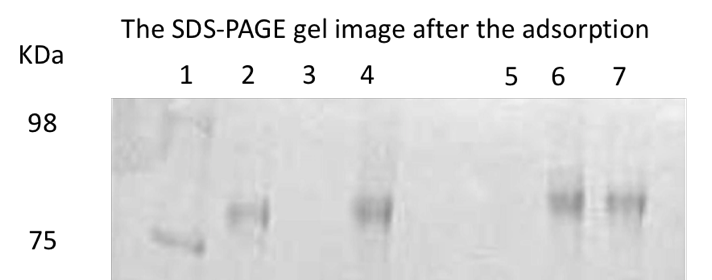


**Supplementary Figure 1: Evaluation of the stability of the – Cy5-Bm86 after adsorption** onto Rho- SV-140-C_18_, Lane 1 – marker, lane 5 – Cy5-Bm86/Rho-SV-140-C_18_ Supernatant, Lane 6 – Cy 5 Bm86/ Rho- SV-140-C_18_ pellet and lane 7 – Bm86 Protein 2mg.


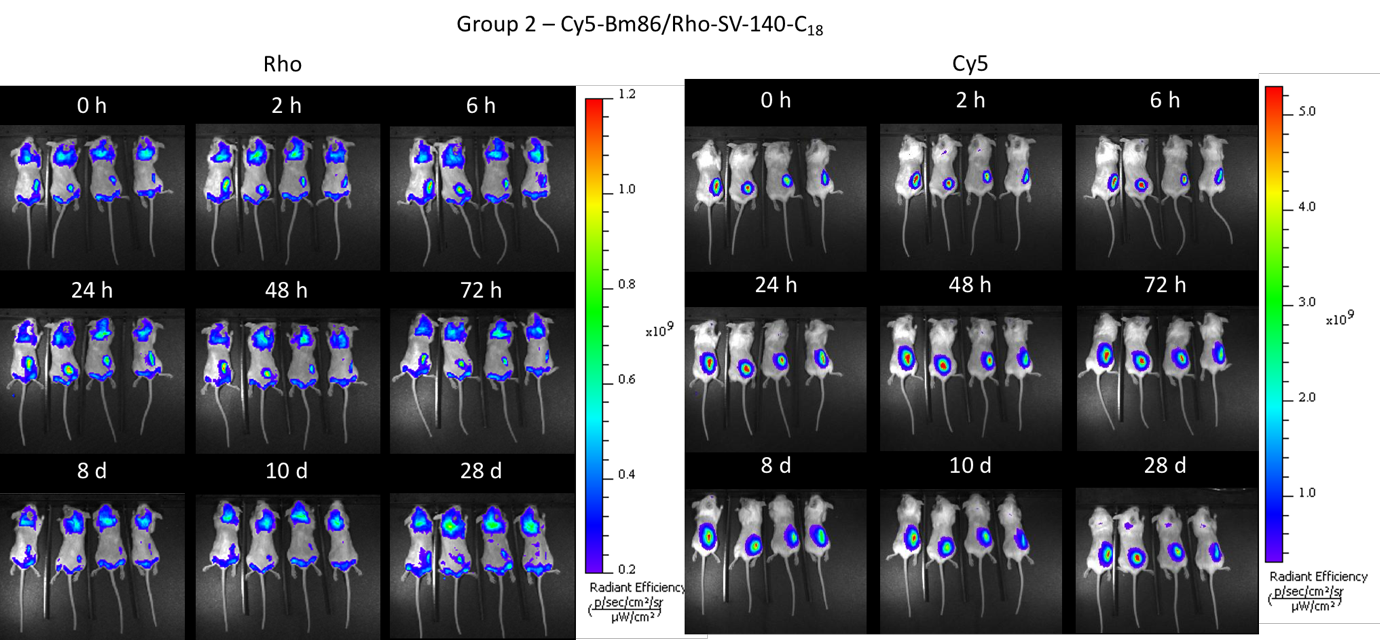


**Supplementary Figure 2:** Live Fluorescence images obtained from imaging BALB/c mice post one subcutaneous injection of Cy5-Bm86 loaded on Rho-SV-140-C_18_ (Group 2) at 0h, 2h, 6h, 24h, 48h, 72h, day-8, day-10 and day-28. The Rhodamine and Cy5 channel show that all of the mice showed a large amount of Rhodamine signal associated with the silica vesicles as well as the Cy5 signal associated to the antigen located at the injection site, suggesting accumulation of the vesicles at the site of injection.


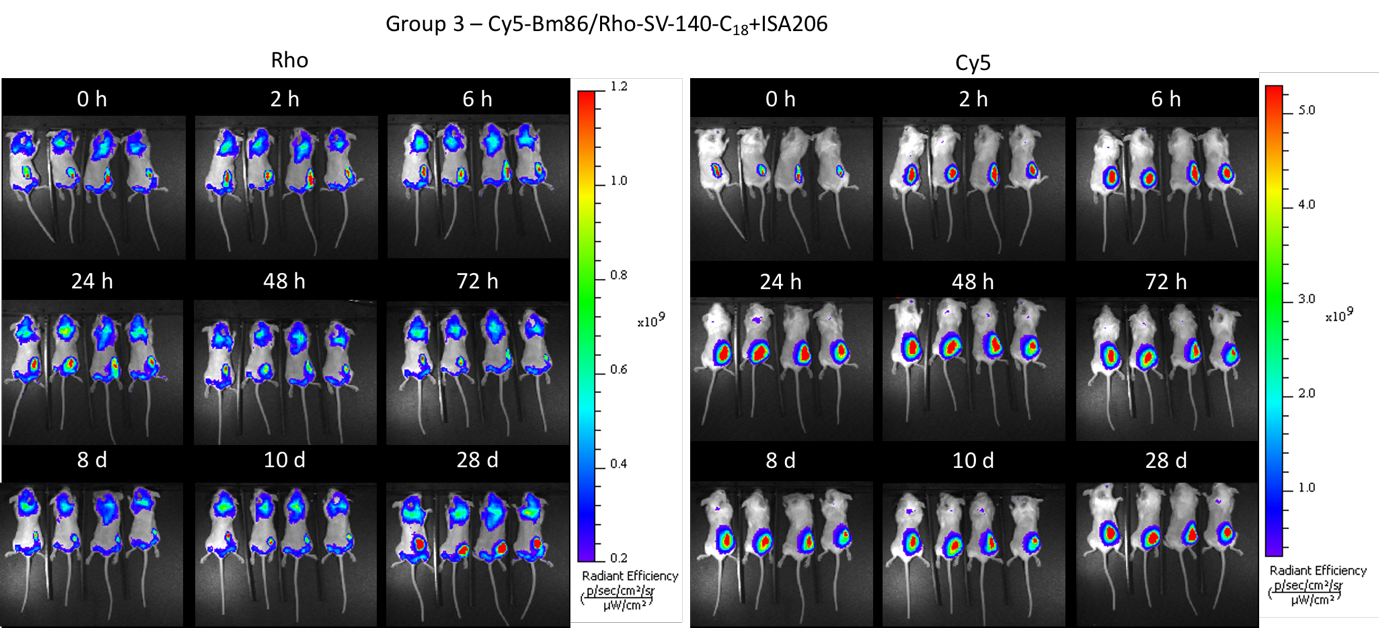


**Supplementary Figure 3:** Live Fluorescence images obtained from imaging BALB/c mice post one subcutaneous injection of Cy5-Bm86 loaded on Rho-SV-140-C_18_ plus ISA206 (Group 3) at 0h, 2h, 6h, 24h, 48h, 72h, day-8, day-10 and day-28. The Rhodamine and Cy5 channel show that all of the mice showed a large amount of Rhodamine signal associated with the silica vesicles as well as the Cy5 signal associated to the antigen located at the injection site, suggesting accumulation of the vesicles at the site of injection.


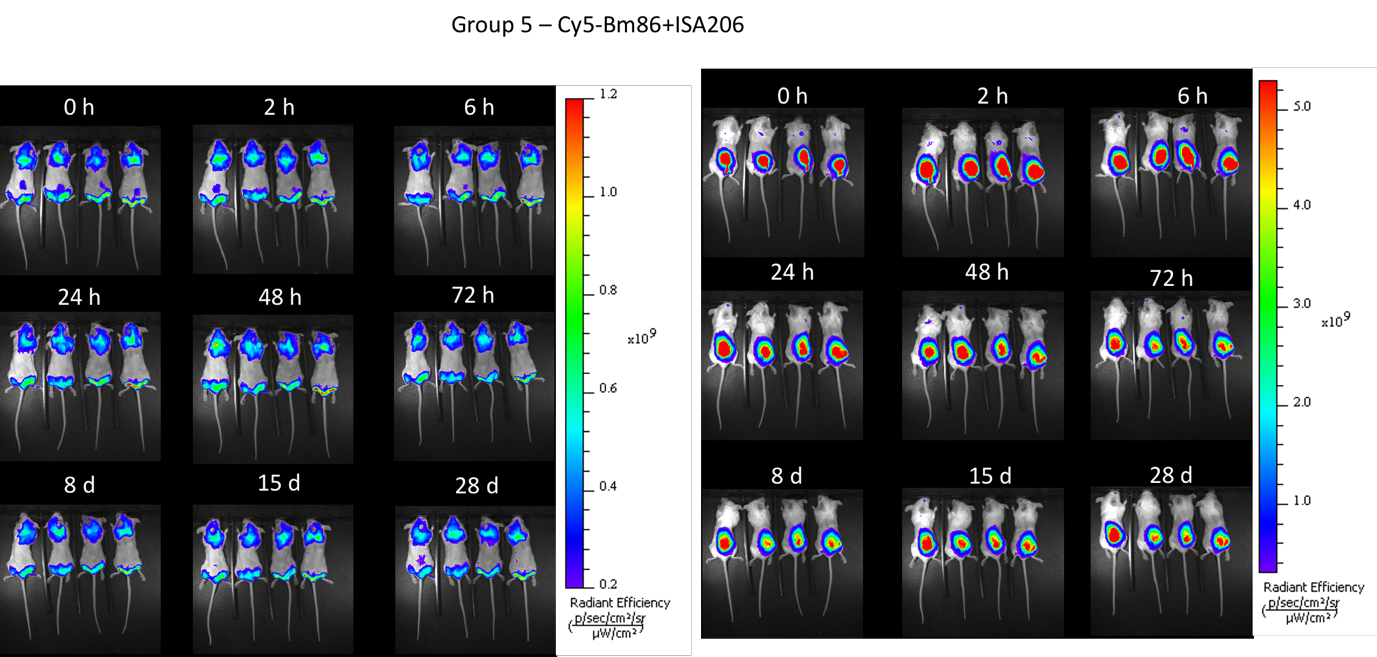


**Supplementary Figure 4:** Live Fluorescence images obtained from imaging BALB/c mice post one subcutaneous injection of Cy5-Bm86 plus ISA206 (Group 5) at 0h, 2h, 6h, 24h, 48h, 72h, day-8, day-10 and day-28. All of the mice showed a large amount of Cy5 signal associated with the antigen located at the injection site, suggesting accumulation of the antigen at the site of injection similar to the group 2 and 3 animals.


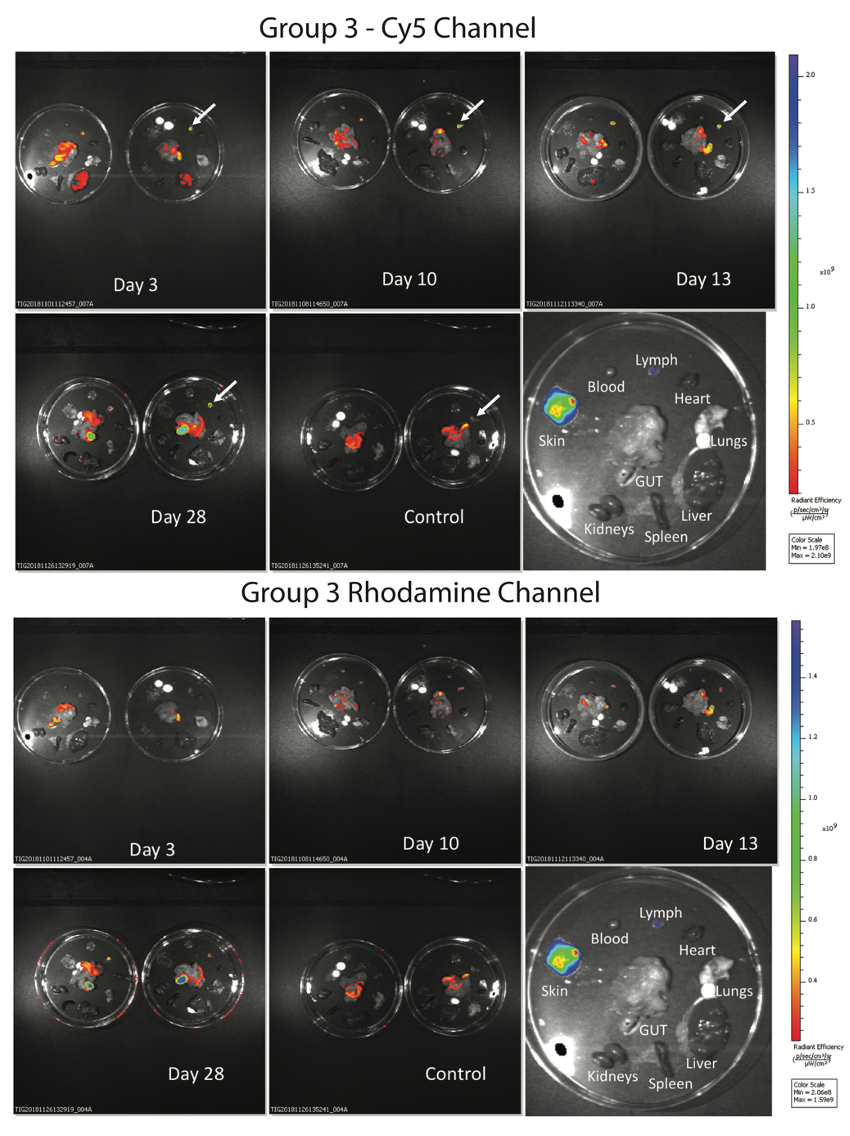


**Supplementary Figure 5: *Ex vivo Fluorescence images*** of the excised organs of BALB/c mice (n=2, at each timepoint) on day 3, day 8 and day 28 after subcutaneous (s.c.) injection for Group 3. Mice (n=2) showed a large amount of Cy 5 signal at the site of injection and lymph node at all the time points.
